# Complications of degenerative lumbar spondylolisthesis and stenosis surgery in patients over 80 s: comparative study with over 60 s and 70 s. Experience with 678 cases

**DOI:** 10.1007/s00701-022-05118-9

**Published:** 2022-02-09

**Authors:** Enrico Aimar, Guglielmo Iess, Federica Mezza, Paolo Gaetani, Alberto Luca Messina, Andrea Todesca, Fulvio Tartara, Giovanni Broggi

**Affiliations:** 1 Department of Neurosurgery, IRCCS Istituto Ortopedico Galeazzi, Milan, Italy; 2 Columbus Clinic Center, Milan, Italy; 3 Department of Vertebral Surgery, Istituto Di Cura Città Di Pavia, Pavia, Italy; 4 Department of Neurosurgery, Fondazione IRCCS Istituto Neurologico Carlo Besta, University of Milan, Milan, Italy; 5 Università Degli Studi Di Milano, Milan, Italy; 6 Department of Economics, University of California, Los Angeles, CA USA; 7 IRCCS Istituto Neurologico Mondino, Pavia, Italy

**Keywords:** Degenerative spondylolisthesis, Lumbar fusion, Elderly, Over 80, Complication rates, Postoperative morbidity, Risk factors

## Abstract

**Purpose:**

Degenerative spondylolisthesis (DS) is a debilitating condition that carries a high economic burden. As the global population ages, the number of patients over 80 years old demanding spinal fusion is constantly rising. Therefore, neurosurgeons often face the important decision as to whether to perform surgery or not in this age group, commonly perceived at high risk for complications.

**Methods:**

Six hundred seventy-eight elder patients, who underwent posterolateral lumbar fusion for DS (performed in three different centers) from 2012 to 2020, were screened for medical, early and late surgical complications and for the presence of potential preoperative risk factors. Patients were divided in three categories based on their age: (1) 60–69 years, (2) 70–79 years, (3) 80 and over. Multiple logistic regression was used to determine the predictive power of age and of other risk factors (i.e., ASA score; BMI; sex; presence or absence of insulin-dependent and -independent diabetes, use of anticoagulants, use of antiaggregants and osteoporosis) for the development of postoperative complications.

**Results:**

In univariate analysis, age was significantly and positively correlated with medical complications. However, when controls for other risk factors were added in the regressions, age never reached significance, with the only noticeable exception of cerebrovascular accidents. ASA score and BMI were the two risk factors that significantly correlated with the higher numbers of complication rates (especially medical).

**Conclusion:**

Patients of different age but with comparable preoperative risk factors share similar postoperative morbidity rates. When considering octogenarians for lumbar arthrodesis, the importance of biological age overrides that of chronological.

**Supplementary Information:**

The online version contains supplementary material available at 10.1007/s00701-022-05118-9.

## Introduction

Degenerative spondylolisthesis (DS) is a pathological condition caused by degenerative alterations in the spine in which one vertebral body is displaced over the one below, and it is commonly associated with spinal stenosis [2, 33].

The first clinical case of spondylolisthesis has been described in Europe in 1782 by a Belgian obstetrician (Herbiniaux) who first named this phenomenon “spondyloptosis,” and depicted this pathological condition as a mechanical hinderance to delivery which reduced the diameter of the pelvic inlet [12]. Nonetheless, the term “spondylolisthesis” was coined for the first time only in 1960 by Newman and Stone [8, 19].

DS results primary from arthritis of the facet joints that thwarts motion of the joint and leads to stress and instability. This, in turn, provokes lessening of the ligamentum flavum, degenerative instability, and consequent anterior displacement of the vertebrae [9, 27].

Both surgical and nonoperative treatments are possible. The former usually involves various types of fusion and stabilization techniques, of which none has yet proven to be superior [5, 18, 34]. Despite the progress made in our understandings of the disease, it has still to be fully established when (and possibly also whether) surgical intervention is superior to conservative treatment [2, 7, 11].

Nevertheless, the number of annual elective lumbar fusions has been on the rise in the last decades with a considerable upward trend of a 118% increase from 1998 to 2014 [29].

The reasons behind such steep increase are multiple and include broader surgical indications, shorter operative durations and significant technological improvements. Moreover, the greater life expectancy of the population and the decrease of post-surgical complication rates have led a growing number of elderlies with DS in their eighties to demand for spinal fusion [15, 23, 29]. It becomes therefore essential to balance benefits and potential complications in these age categories commonly considered as fragile and at risk for such invasive procedures.

Is spinal fusion in patients approaching their nine’s, eight’s and seventh’s decades of life equally safe? Are there some risk factors (i.e., obesity, insulin-dependent and insulin-independent diabetes, use of antiaggregant or anticoagulant therapy, osteoporosis, and ASA IV) in this population that drive the rates of post-operative medical, early surgical, and late surgical complications?

### Aim of this study

Our primary objective in this paper was to evaluate whether medical and surgical risks are significantly different between patients in their sixties, seventies, and eighties undergoing open posterolateral arthrodesis with associated spinal decompression. We hypothesize that complication rates between these different age groups are not significantly different as long as premorbid profiles are similar.

## Material and methods

### Inclusion criteria, surgical technique, and parameters analyzed

In this retrospective study, clinical data were gathered and analyzed from 678 consecutive cases of patients (250 males and 428 females) who underwent arthrodesis using an open posterolateral instrumentation with pedicle screw fixation and laminectomy for spinal decompression. Surgeries were performed between January 2012 and May 2020 in IRCCS Istituto Ortopedico Galeazzi (Gruppo ospedaliero San Donato), Milan, Italy, Columbus Clinic Center, Milan, Italy, and Istituto di Cura Città di Pavia (Gruppo ospedaliero San Donato), Pavia, Italy. Diagnosis was formulated by the first author (E.A.) who also carried out the surgical procedure as the first operator. Data were collected by reviewing hospital charts, admission letters, and follow-up visits. Mean follow-up time was 4 years and 9 months.

Inclusion criteria were 1 or 2 levels of spondylolisthesis, evidence of Schizas grade C or D spinal stenosis on MRI, neurogenic claudication, absence of degenerative scoliosis > 5°, and vertebral instability documented by a dynamic orthostatic X-rays radiography.

Patients that were taking anticoagulant or antiaggregant therapies before surgery underwent a cardiological examination to evaluate the most appropriate Low Molecular Weight Heparin (LMWH) bridging regimen.

Generally, individuals taking warfarin discontinued the drug 5 days prior to the operative intervention with an international normalized ratio’s target inferior to 1.5. Likewise, antiplatelet therapies with either aspirin or plavix were stopped 5 days before fusion and stabilization. Differently, oral direct factor Xa inhibitors (for example rivaroxaban, apixaban, and edoxaban) were suspended 48 h ahead of the surgical procedure.

Usually, all patients under anticoagulant or antiplatelet therapy transited to LMWH; the latter, however, was almost invariably held for 12 to 24 h before surgery. Nevertheless, special situations which required different bridging regimens were adjusted on an individual basis.

All patients underwent perioperative antibiotic therapy which consisted of a standard protocol with 2 g of intravenous cefazolin administrated 2 h before and 6 h after the surgical procedure (except for patients with known allergies to the aforementioned antibiotic therapy).

In every individual, a urinary catheter was placed and subsequently removed after the patient started autonomous mobilization.

Information we searched for (other than age) included postoperative complications and hypothetical preoperative risk factors (Table 1).

**Table 1:** Complete list of risk factors and outcome parameters analyzed in the article

Risk factors	Continuous variables	• Age
		• Body mass index (BMI)
	Categorical variables	• ASA
		• Insulin dependent (ID) Diabetes
		• Non-insulin dependent (NID) Diabetes
		• Use of antiaggregants
		• Use of anticoagulants
		• Osteoporosis
		• Sex
Complications	Medical	• Urinary tract infections (UTI)
		• Pneumonia
		• Deep venous thrombosis (DVT)
		• Cerebrovascular accidents
		• Cardiological complications
	Early surgical	• CSF leak
		• Wound dehiscence
		• Haematoma
		• Radicular post decompressive deficit
	Late surgical	• Spondylodiscitis
		• Device mobilization/rupture
		• Adjacent segment pathology

More specifically, postoperative complications comprised of the following: (1) Early surgical complications which occurred during the first 15 days from the operation (cerebrospinal fluid (CSF) leak, wound dehiscence, post-surgical hematoma that necessitated incision and drainage, and radicular post-decompressive deficit); (2) Late surgical complications that occurred 15 days after surgery (spondylodiscitis, device mobilization/rupture, and adjacent segment pathology); (3) Medical complications within 3 months from the day of surgery (urinary tract infection (UTI), pneumonia, deep vein thrombosis (DVT), cerebrovascular accident, and cardiological complications).

Risk factors considered included the following: (1) ASA class, (2) BMI, (3) osteoporosis, (4) use of anticoagulants, (5) use of platelet antiaggregants, (6) non-insulin dependent (NID) diabetes mellitus, (7) insulin dependent (ID) diabetes mellitus, (8) sex.

### Statistical analysis

Frequency and percentages were utilized to describe categorical variables while mean, ranges, and standard deviations were used for continuous.

Two cases with missing values of one or more predictors were excluded from the regression analysis.

Pearson’s chi-square test was used to compare frequencies between two or more categorical parameters. For every complication considered, we utilized multiple logistic regressions to predict its specific probability of development after surgery. Eight of the predictor variables were categorical while BMI was the only continuous one. Age was considered categorical and divided into 3 groups: (1) patients 60–69 years old, (2) patients 70–79 years old, (3) patients 80 and older. Regression assumptions were not violated and this was checked through tests. All predictors were tested for multicollinearity by calculating their variance inflation factors which were always deemed as acceptable.

Full model was compared with a model of intercept only by means of chi-square test. Furthermore, Hosmer and Lemeshow test was also performed to assure proper goodness-of-fit. For each of the predictors, standardized logistic regression coefficients and *p* values were calculated.

All *P* values reported are two-tailed, and *P* < 0.05 was considered statistically significant.

Computations and graphics were made using R (version 4.1.0) and SPSS (IBM Corp. Release, IBM SPSS Statistics for macOS, Version 26.0).

## Results

**Graph 1:**
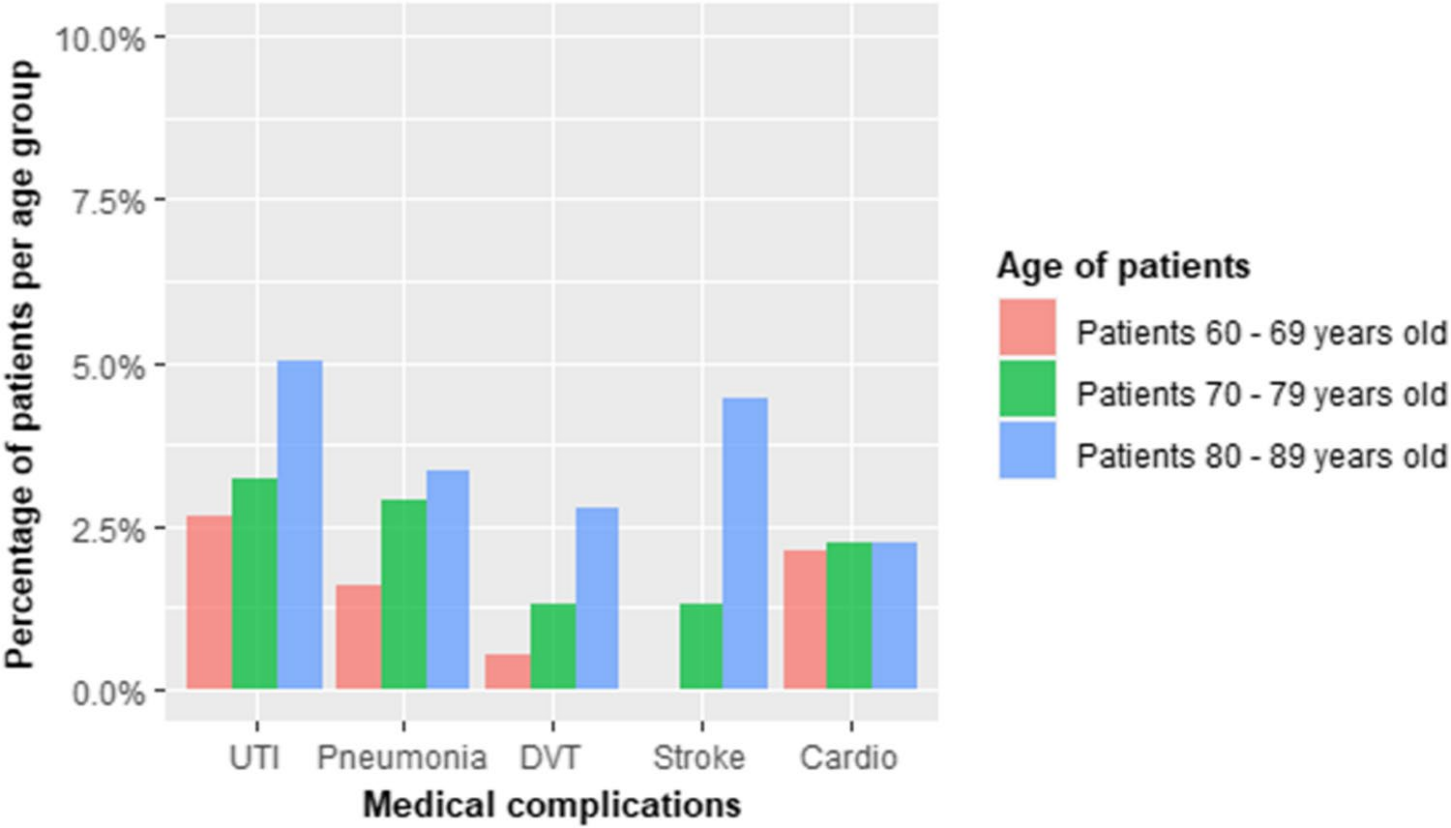
Distribution of different medical complications weighted by specific age groups. *UTI*, urinary tract infections; *DVT*, deep vein thrombosis

**Graph 2:**
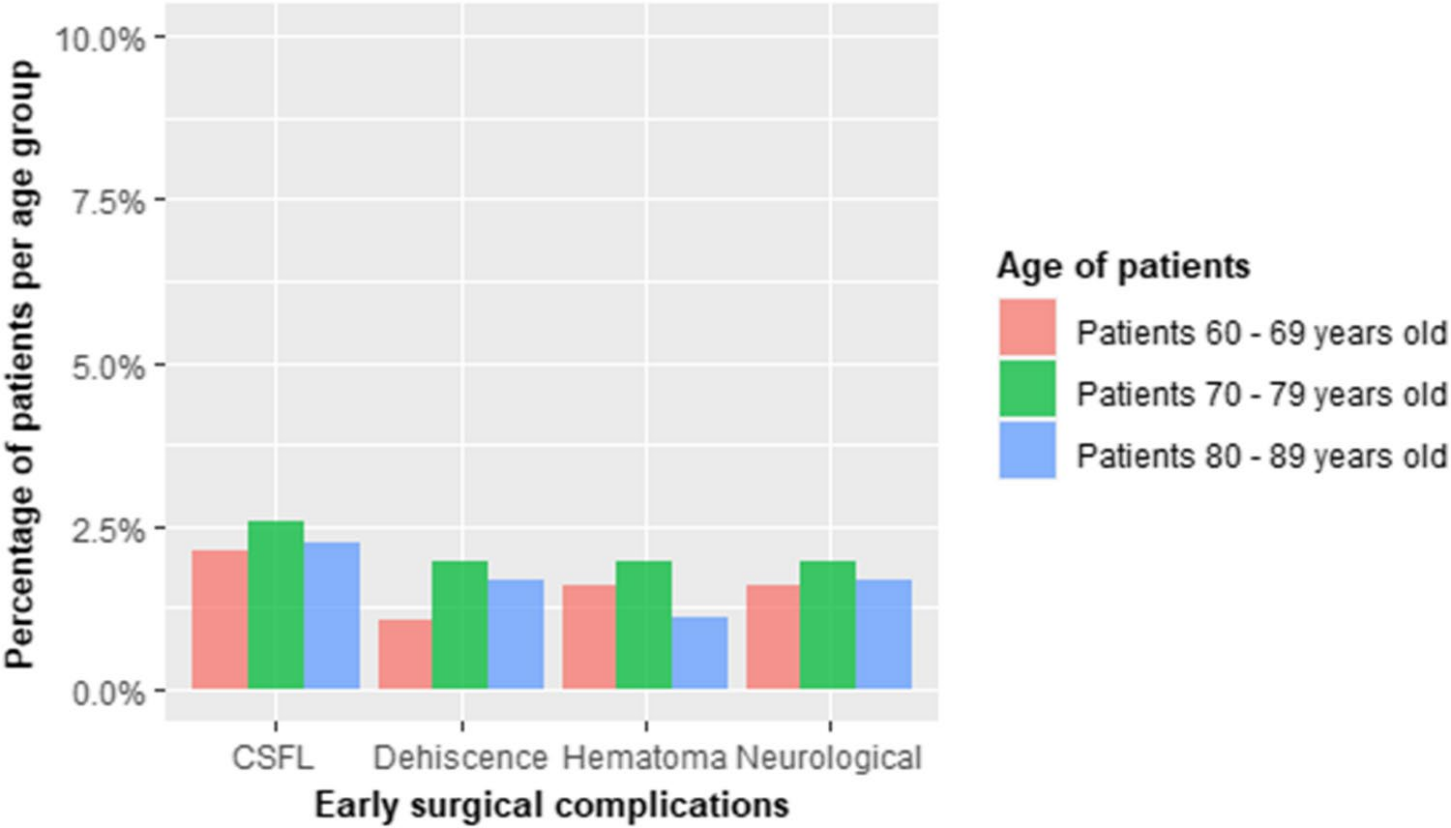
Distribution of different early surgical complications weighted by specific age groups. *CSFL*, cerebro spinal fluid leak. Neurological complications are represented by radicular post-decompressive deficits

**Graph 3:**
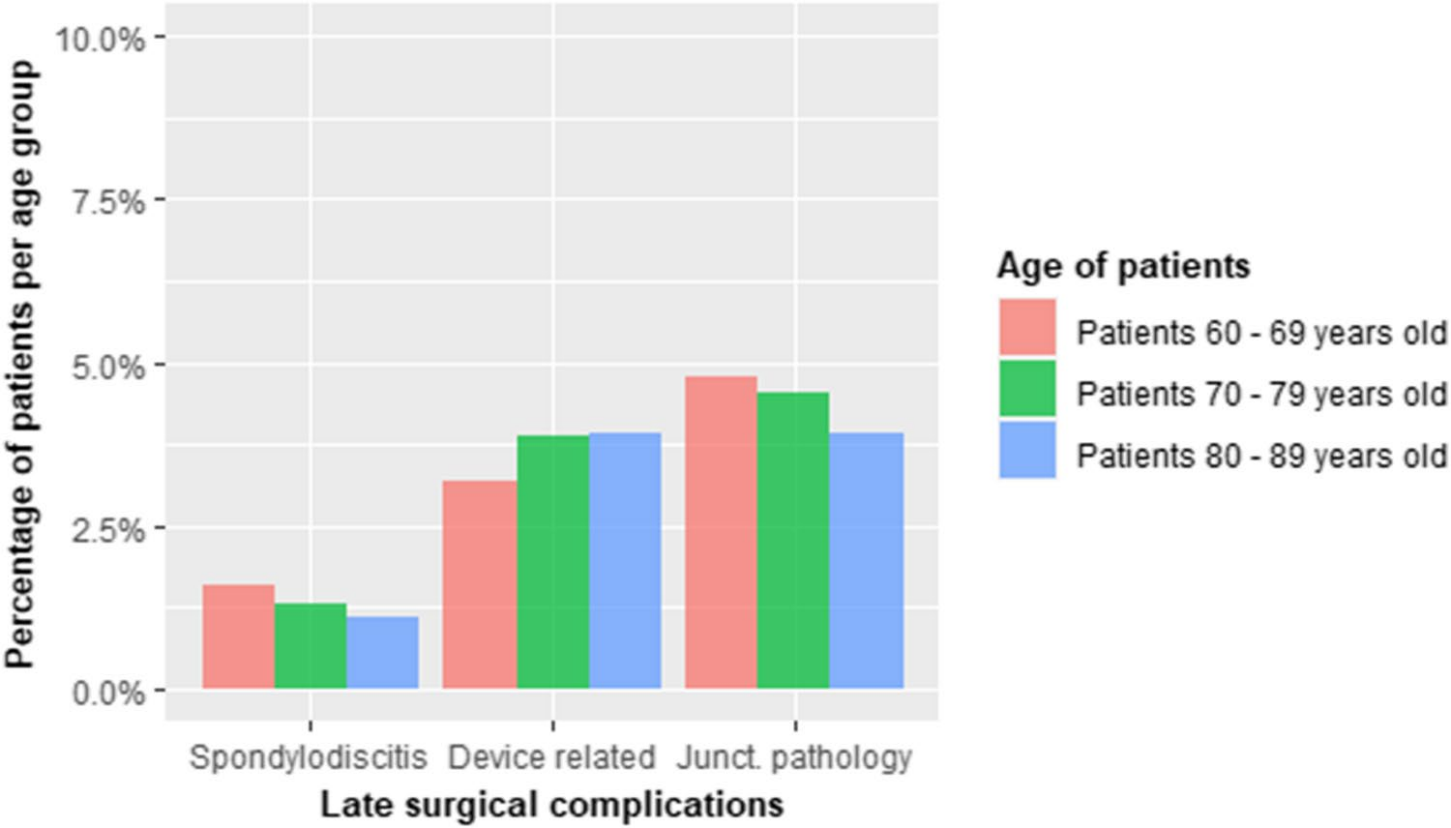
Distribution of different late surgical complications weighted by specific age groups. Device-related complications include pedicle screw fracture or loosening, hardware failure, implant migration, or deformation

Table 2 and Graphs 1, 2, and 3 illustrate descriptive statistics. Our cohort averaged 74.61 years (*SD* = 6.47) and included 188 patients between 60 and 69 years of age, 309 between 70 and 79, and 179 between 80 and 89.

**Table 2:** Descriptive statistics representing a complete list of the risk factors analyzed in the article. Means with their respective standard deviations are used to describe continuous variables, while frequencies and percentages for categorical

Risk factors	
• Age (mean, SD, range)	74.61 ± 6.47, 60–89
• BMI (mean, SD, range)	26.06 ± 2.74, 19–35
• ASA (*n*, %)	ASA 1 = 104 (15%); ASA 2 = 250 (37%); ASA 3 = 221 (33%); ASA 4 = 100 (15%)
• Insulin dependent diabetes (*n*, %)	43 (6%)
• Non-insulin dependent diabetes (*n*, %)	60 (9%)
• Use of antiaggregants (*n*, %)	165 (24%)
• Use of anticoagulants (*n*, %)	60 (9%)
• Osteoporosis (*n*, %)	176 (26%)
• Sex (*n*, %)	*M* = 250 (37%); *F* = 428 (63%)

Seventy-nine medical complications were recorded: 24 urinary tract infections, 18 lobar pneumonias, 10 deep vein thrombosis (2 of which were further complicated by pulmonary embolism), 12 cerebrovascular accidents (8 transient ischemic attacks, 4 ischemic strokes, one of which resulted in death), and 15 cardiological complications (8 atrial fibrillations requiring pharmacological treatment, 7 myocardial infarctions including 5 treated with coronary angioplasty and stent placement and 1 fatality).

Fifty-two early surgical complications were also encountered that presented the following distributions: 16 CSF leaks (seven of which required surgical revision), 11 wound dehiscences (nine necessitating reoperation); 13 postsurgical hematomas (all subsequently incised and drained), 12 radicular post decompressive deficits (6 of which did not report any further neurological recovery).

Total late surgical complications were 63 (12 spondylodiscitis, 21 device mobilizations/screw fractures, and 30 adjacent segment diseases). Table 3 summarizes complications’ distribution with their respective percentages.

**Table 3:** Significant correlations between total medical, early surgical, and late surgical complications and risk factors. *BMI*, body mass index; *DVT*, deep vein thrombosis; *UTI*, urinary tract infections; *ID*, insulin dependent; *NID*, non-insulin dependent. * Negative (inverse) correlation

	*P* < 0.05	*P* < 0.01	*P* < *0.001*
Medical complications	NID diabetes*		BMI, ASA, anticoagulant therapy, ID diabetes
Early surgical complications	Antiaggregant therapy		
Late surgical complications	Osteoporosis		

The different distributions and means of the hypothetical risk factors are analyzed and illustrated in Table 2. More specifically, there were 104 patients in ASA class 1 (15.41%), 250 ASA class 2 (37.04%), 221 with ASA 3 (32.74%), and 100 in a constant threat of life (ASA 4, 14.81%).

Sixty elderlies were under anticoagulant therapy, while 165 under antiaggregant. BMI averaged 26.06 (*SD* = 2.74). One hundred seventy-six individuals suffered from osteoporosis, while 103 had diabetes mellitus (60 of which were insulin-independent and 43 insulin-dependent).

Statistical analysis demonstrated that the percentage of individuals with ASA 4 and the percentage of patients taking anticoagulant therapy was significantly lower between individuals in their sixties and seventies with respect to those in their eighties (8%, 14.3%, and 23%, respectively for ASA 4 (*Χ*^2^ (2) = 39.357, *P* < 0.001) and 16.7%, 41.7%, and 41.7%, respectively for use of anticoagulants (*Χ*^2^ (2) = 8.911, *P* = 0.012)) which probably accounts for the higher absolute number of medical complications found in the latter group (*Χ*^2^ (2) = 9.041, *P* = 0.003).

A high BMI raises considerably chances of suffering from medical complications, irrespective of age groups (*B* = 0.366, Wald = 37.701, *P* < 0.001).

Taking antiaggregant therapy did not increase the risk of developing medical complications in this type of surgery. It may have only a weak correlation with overall early surgical complications (*B* = 0.910, Wald = 5.895, *P* = 0.015), especially for what concerns wound dehiscence (*B* = 1.754, Wald = 4.684, *P* = 0.030). Similarly, osteoporosis was the only weak significant risk factor that correlated with late surgical complications (*B* = 0.712, Wald = 3.874, *P* = 0.049).

Importantly, when controls for the other variables were added to the regression, age was never a significant predictor for any of the above listed complications except cerebrovascular diseases (Table 3 and Table 4) which on the contrary were significant at *P* = 0.005 (*B* = 1.745, Wald = 7.715).

**Table 4:** Correlations between single complications and risk factors that reached statistical significance. *DVT*, deep vein thrombosis; *UTI*, urinary tract infections; *ID*, insulin dependent

	*P* < 0.05	*P* < 0.01	*P* < *0.001*
Medical complications			
UTI	ASA		BMI
Pneumonia	ASA	BMI	
DVT	ASA, anticoagulant therapy		
Cerebrovascular accidents		Age	BMI
Cardiological complications	ASA	ID diabetes	
Early surgical complications			
Fistula			
Wound dehiscence	Antiaggregant therapy		
Hematoma			
Radicular postdecompressive			
Late surgical complications			
Spondylodiscitis		BMI, ASA	
Device mobilization/rupture		BMI	
Adjacent segment pathology			

Conversely, other risk factors such as ASA (*B* = 0.888, Wald = 15.019, *P* < 0.001), ID diabetes (*B* = 1.734, Wald = 13.896, *P* < 0.001), and anticoagulant use (*B* = 1.696, Wald = 16.556, *P* < 0.001) positively correlated with medical complication rates. Interestingly, NID diabetes had a negative correlation with the latter (*B* =  − 1.051, Wald = 4.090, *P* = 0.043).

Considered all together, early surgical complications were not different between the three age groups. Post-surgical CSF leaks, hematomas, and neurological deficits were not determined by any other risk factors.

Finally, in our study, ASA and BMI were the two risk factors that positively correlated with the highest number of complications; more specifically, both were associated with UTI, pneumonia, cerebrovascular accidents, and spondylodiscitis, while ASA was with TVP alone and BMI with device mobilization alone as well.

## Discussion

In surgical procedures, postoperative morbidity and mortality is historically believed to increase progressively with growing age [6, 17, 21, 31]. Yet, this correlation does not apply uniformly for all types of surgery, and some pose the elderly at higher risk for complications than others [10].

Today, as global population ages (in 2050, the life expectancy is believed to raise to 86.6 and 81.1 years for women and men, respectively) the increased prevalence of degenerative spondylolisthesis is accelerating the demand for spinal fusion in patients 80 years or older, once considered unsuitable candidates [30]. Although elective spinal surgery is often not perceived as lifesaving, withholding it (and therefore leaving conditions like spinal stenosis, disc herniations or segmental instability untreated) has relevant implications for the quality of life of patients. Moreover, due to the significant morbidity of DS, the load on the healthcare is destined to rise substantially in the future.

Predicting preoperative risk is surely an imperfect science that integrates different perspectives also resulting from different needs (i.e., those of the surgeon, of the anesthesiologist, and sometimes of other physicians). Nonetheless, in the last 20 years, a growing number of physicians have been attempting to elaborate different frailty indexes in the process of preoperative risk stratification.

Although many definitions are used, frailty is commonly considered a geriatric syndrome that exposes older adults at a high risk of devastating decline in health and function and predisposes the surgical patient to higher rates of postoperative complications [32]. In literature, 11 different indexes can be found attempting to quantitatively assess frailty in patients undergoing spinal surgeries, and that therefore try to predict the occurrence of postoperative complications. The superiority of one vs the other is a matter of debate, and it is not the intention of the authors to elaborate on the topic [32]. The approach of this study was to test the predictive power of the most commonly diffuse medical conditions on medical, early surgical, and late surgical complications.

Despite the fact that most investigators have limited their studies to patients with less than 70 years of age, the efficacy of spinal fusion in the elderlies has been demonstrated by various retrospective studies [4, 14].

Nonetheless, few authors have analyzed complication rates of spinal surgery in general (i.e., not exclusively restricted to arthrodesis) in patients over 80, and those who did, not only have reported conflicting results but also with small sample sizes; however, spinal fusion is a more invasive surgery with respect to decompression and microdiscectomy, and in line with what other investigators have reported so far, our statistical analysis indicated that octogenarians had significantly higher rates of complications compared to individuals in their seventies or sixties for both medical and surgical complications [3, 17, 22, 26].

But when controls for all other risk factors were added in the analysis, age suddenly was never a significant factor in predicting any of the complications examined. The only noticeable exception was represented by cerebrovascular accidents for which age did exhibit a positive correlation. Perioperative strokes are mostly ischemic and embolic in nature [28]. Surgical trauma with associated tissue injury is a well-known cause of hypercoagulability, but the mechanism by which age probably increases the risk of strokes is constituted by the decreased cerebrovascular reserve and by the many pathological coexisting conditions (i.e., diabetes, hypertension, carotid stenosis, and BPCO) that occur with a higher frequency in older people [28]. Lately, a protective effect on postoperative cerebrovascular accidents has been identified in the use of volatile anesthesia [24].

Likewise, it is also interesting to note that as insulin-dependent diabetes emerged as a strong predictor of medical (mostly cardiological) complications, non-insulin diabetes did not (and even appeared to have a weak inverse correlation). Such impressive discrepancy may be explained by the protective effect (which lately emerged) exerted by metformin, the most-commonly prescribed drug for this condition worldwide, on reducing risk-adjusted mortality, intensive care unit length of stay, and readmission following surgeries [16, 25].

Besides the effect of age, our findings also highlight the powerful impact that a high BMI and high ASA class exert on medical and late surgical complication rates, thus representing the two most influential predictors. In our previous paper, we demonstrated how these two parameters negatively influenced clinical outcome in patients undergoing simple decompressive surgery for spinal stenosis [1]. Our findings in this paper confirm how these two fundamental parameters should be routinely integrated in the process of selection of candidates for lumbar surgery.

### Strengths and limitations

With 678 cases of lumbar open posterolateral fusions, our study represents one of the largest multi-center studies evaluating complications of spinal arthrodesis in the elderlies, and especially the one with the highest cohort of cases (179) of patients with more than 80 years. Its considerable mean follow-up time (4 years and 9 months) allowed for a complete assessment of a wide range of both short- and long-term medical and surgical complications. Furthermore, all surgical procedures have been performed by the same surgeon (E.A.), making our study more robust to performance bias.

Nevertheless, some study limitations need to be pointed out. First of all, this study only includes surgically treated patients; therefore, there is a chance that patients with high morbidity profiles may have been excluded from the analysis, resulting in a surgical selection bias which may have hampered the results. Secondly, because of the retrospective nature of the study, it is possible that some of the complications were underreported. The rates of CSF leaks and hardware failure, for example, were low compared to literature [13, 20, 35]. Hindsight bias and recall bias might have influenced the frequencies reported. Thirdly, the risk factors we analyzed are only some among the most widely diffused medical conditions encountered by spinal surgeons in their daily practice, thence do not necessarily represent a complete list of variables that may influence complication rates in the elderlies undergoing lumbar fusions. Other less diffused risk factors may be implicated as well.

Lastly, using a binary classification system to measure complications’ outcomes, although offering greater versatility when interpreting the results, decreases the capacity to detect relationships.

## Conclusions

In patients undergoing open lumbar posterolateral arthrodesis and decompression (with vertebral instability documented by a dynamic orthostatic X-rays radiography, 1 or 2 levels, no scoliosis greater than 5°, and Schizas C or D spinal grade of compression) with similar risk factors, the rates of postoperative complications do not increase with increasing age. Instead, complication rates rise uniformly throughout different age groups, along with the presence of important pre-operative risk factors: ASA class IV, high BMI, insulin-dependent diabetes mellitus, and use of anticoagulants for what concerns medical complications; ASA class IV and elevated BMI for spondylodiscitis and elevated BMI with regard to device mobilization/rupture.

Healthy octogenarians are at lower risk for medical and surgical complications than individuals in their sixties with one, or more, risk factors. When selecting the ideal candidates for lumbar fusion surgery, the importance of biological age outweighs that of chronological.

## Supplementary Information

Below is the link to the electronic supplementary material.

Supplementary file1 (PDF 2304 KB)
