# Supplementary material for: Complications of degenerative lumbar spondylolisthesis and stenosis surgery in patients over 80 s: comparative study with over 60 s and 70 s. Experience with 678 cases
Source: Acta Neurochir (Wien). 2022 Feb 9;164(3):923–31. doi: 10.1007/s00701-022-05118-9 (PMC8913488; doi:10.1007/s00701-022-05118-9)
Supplement: Supplementary file 1 — Supplementary file1 (PDF 2304 KB) [file 701_2022_5118_MOESM1_ESM.pdf]

Adis | Bohn Stafleu van Loghum | palgrave macmillan | Springer

Change of authorship request form (pre-acceptance)

Please read the important information on page 4 before you begin

This form should be used by authors to request any change in authorship including changes in corresponding authors. Please fully complete all sections. Use black ink and block capitals and provide each author's full name with the given name first followed by the family name.

Please note: In author collaborations where there is formal agreement for representing the collaboration, it is sufficient for the representative or legal guarantor (usually the corresponding author) to complete and sign the Authorship Change Form on behalf of all authors.

**Manuscript ID no. in case of unpublished manuscript:**	ANCH-D-21-01785R1
**DOI in case of published manuscript:**	
**ISBN (for books):**	

Section 1: Please provide the current title of manuscript
(For journals: Please provide the manuscript ID, title and/or DOI if available.)
(For books: Please provide the title, ISBN and/or DOI if available.)

Title: COMPLICATIONS OF DEGENERATIVE LUMBAR SPONDYLOLISTHESIS AND STENOSIS SURGERY IN PATIENTS OVER 80S: COMPARATIVE STUDY WITH OVER 60S AND 70S. EXPERIENCE WITH 678 CASES

Section 2: Please provide the previous authorship, in the order shown on the manuscript before the changes were introduced. Please indicate the corresponding author by adding (CA) behind the name.

	First name(s)	Family name	ORCID or SCOPUS id, if available
1st author	ENRICO	AIMAR	
2nd author	GUGLIELMO	IESS	
3rd author	FEDERICA	MEZZA	
4th author	PAOLO	GAETANI	
5th author	ALBERTO LUCA	MESSINA	
6th author	ANDREA	TODESCA	
7th author	FULVIO	TARTARA	

Please use an additional sheet if there are more than 7 authors.

SPRINGER NATURE Springer Nature is one of the world's leading global research, educational and professional publishers, created in May 2015 through the combination of Nature Publishing Group, Palgrave Macmillan, Macmillan Education and Springer Science+Business Media. ---

Adis Bohn Stafleu van Loghum palgrave macmillan Springer

Change of authorship request form (pre-acceptance)

Section 2: Please provide the previous authorship, in the order shown on the manuscript before the changes were introduced. Please indicate the corresponding author by adding (CA) behind the name.

	First name(s)	Family name	ORCID or SCOPUS id, if available
8th author	GIOVANNI	BROGGI	

SPRINGER NATURE Springer Nature is one of the world's leading global research, educational and professional publishers, created in May 2015 through the combination of Nature Publishing Group, Palgrave Macmillan, Macmillan Education and Springer Science+Business Media. ---

Adis · Bohn Stafleu van Loghum · palgrave macmillan · Springer

Change of authorship request form (pre-acceptance)

Section 3: Please provide a justification for change. Please use this section to explain your reasons for changing the authorship of your manuscript, e.g. what necessitated the change in authorship? Please refer to the (journal) policy pages for more information about authorship. Please explain why omitted authors were not originally included and/or why authors were removed on the submitted manuscript.

DR. BONOMO GIULIO HAS BEEN ADDED AS A CO-AUTHOR AFTER HELPING WITH THE REVISION' AND RESPONSE TO REVIEWERS' PROCESSES

Section 4: Proposed new authorship. Please provide your new authorship list in the order you would like it to appear on the manuscript. Please indicate the corresponding author by adding (CA) behind the name. <u>If the corresponding author has changed, please indicate the reason under section 3.</u>

	First name(s)	Family name (this name will appear in full on the final publication and will be searchable in various abstract and indexing databases)
1st author	ENRICO	AIMAR
2nd author	GUGLIELMO	LESS
3rd author	FEDERICA	MEZZA
4th author	PAOLO	GAETANI
5th author	ALBERTO LUCA	MESSINA
6th author	ANDREA	TODESCA
7th author	FULVIO	TARTARA

Please use an additional sheet if there are more than 7 authors.

SPRINGER NATURE Springer Nature is one of the world's leading global research, educational and professional publishers, created in May 2015 through the combination of Nature Publishing Group, Palgrave Macmillan, Macmillan Education and Springer Science+Business Media. ---

Adis Bohn Stafleu van Loghum palgrave macmillan Springer

Change of authorship request form (pre-acceptance)

Section 4: Proposed new authorship. Please provide your new authorship list in the order you would like it to appear on the manuscript. Please indicate the corresponding author by adding (CA) behind the name. <u>If the corresponding author has changed, please indicate the reason under section 3.</u>

	First name(s)	Family name (this name will appear in full on the final publication and will be searchable in various abstract and indexing databases)
8th author	GIULIO	BONOMO
9th author	GIOVANNI	BROGGI

Change of authorship request form (pre-acceptance)

Section 5: Author contribution, Acknowledgement and Disclosures. Please use this section to provide a new disclosure statement and, if appropriate, acknowledge any contributors who have been removed as authors and ensure you state what contribution any new authors made (if applicable per the journal or book (series) policy). Please ensure these are updated in your manuscript - after approval of the change(s) - as our production department will not transfer the information in this form to your manuscript.

New acknowledgements: NONE

New Disclosures (financial and non-financial interests, funding): NONE

New Author Contributions statement (if applicable per the journal policy): DR. BONOMO CONTRIBUTED TO THE MANUSCRIPT DURING THE 'REVISION' AND 'RESPONSE TO REVIEWERS' PROCESSES

State 'Not applicable' if there are no new authors.

Adis | Bohn Stafleu van Loghum | palgrave macmillan | Springer

Change of authorship request form (pre-acceptance)

Section 6: Declaration of agreement. *All* authors, unchanged, new and removed *must* sign this declaration.

(NB: Please print the form, sign and return a scanned copy. Please note that signatures that have been inserted as an image file are acceptable as long as it is handwritten. Typed names in the signature box are unacceptable.) *** Please delete as appropriate. Delete all of the bold if you were on the original authorship list and are remaining as an author.**

	First name	Family name		Signature	Affiliated institute	Date
1st author	ENRICO	AIMAR	I agree to the proposed new authorship shown in section 4 /and the addition/removal*of my name to the authorship list.	[signature]	IRCCS ISTITUTO ORTOPEDICO GALEAZZI	11/1/22
2nd author	GUGLIELMO	IESS	I agree to the proposed new authorship shown in section 4 /and the addition/removal*of my name to the authorship list.	[signature]	FONDAZIONE IRCCS ISTITUTO NEUROLOGICO CARLO BESTA	06/01/2022
3rd author	FEDERICA	MEZZA	I agree to the proposed new authorship shown in section 4 /and the addition/removal*of my name to the authorship list.	[signature]	UNIVERSITY OF CALIFOR-NIA	06/01/2022
4th authors	PAOLO	GAETANI	I agree to the proposed new authorship shown in section 4 /and the addition/removal*of my name to the authorship list.	[signature]	ISTITUTO DI CURA CITTÀ DI PAVIA	10/01/22
5th author	ALBERTO LUCA	MESSINA	I agree to the proposed new authorship shown in section 4 /and the addition/removal*of my name to the authorship list.	[signature]	ISTITUTO DI CURA CITTÀ DI PAVIA	11/01/22
6th author	ANDREA	TODESCA	I agree to the proposed new authorship shown in section 4 /and the addition/removal*of my name to the authorship list.	[signature]	ISTITUTO DI CURA CITTÀ DI PAVIA	10/01/22
7th author	FULVIO	TARTARA	I agree to the proposed new authorship shown in section 4 /and the addition/removal*of my name to the authorship list.	[signature]	ISTITUTO DI CURA CITTÀ DI PAVIA	10/01/21

Please use an additional sheet if there are more than 7 authors.

SPRINGER NATURE Springer Nature is one of the world's leading global research, educational and professional publishers, created in May 2015 through the combination of Nature Publishing Group, Palgrave Macmillan, Macmillan Education and Springer Science+Business Media. —

Scansionato con CamScanner

Adis Bohn Stafleu van Loghum palgrave macmillan Springer

Change of authorship request form (pre-acceptance)

Section 6: Declaration of agreement. *All* authors, unchanged, new and removed *must* sign this declaration.

(NB: Please print the form, sign and return a scanned copy. Please note that signatures that have been inserted as an image file are acceptable as long as it is handwritten. Typed names in the signature box are unacceptable.) *** Please delete as appropriate. Delete all of the bold if you were on the original authorship list and are remaining as an author.**

	First name	Family name		Signature	Affiliated institute	Date
8th author	GIULIO	BONOMO	I agree to the proposed new authorship shown in section 4 **/and the addition/removal*of my name to the authorship list.**		FONDAZIONE IRCCS ISTITUTO NEUROLOGICO CARLO BESTA	
9th author	GIOVANNI	BROGGI	I agree to the proposed new authorship shown in section 4 **/and the addition/removal*of my name to the authorship list.**		FONDAZIONE IRCCS ISTITUTO NEUROLOGICO CARLO BESTA	
10th author			I agree to the proposed new authorship shown in section 4 **/and the addition/removal*of my name to the authorship list.**			

Please use an additional sheet if there are more than 7 authors.

SPRINGER NATURE Springer Nature is one of the world's leading global research, educational and professional publishers, created in May 2015 through the combination of Nature Publishing Group, Palgrave Macmillan, Macmillan Education and Springer Science+Business Media. ---

Adis Bohn Stafleu van Loghum palgrave macmillan Springer

Change of authorship request form (pre-acceptance)

Important information. Please read.

Please return this form, fully completed, to Springer Nature. We will consider the information you have provided to decide whether to approve the proposed change in authorship. We may choose to contact your institution for more information or undertake a further investigation, if appropriate, before making a final decision.

By signing this declaration, all authors guarantee that the order of the authors are in accordance with their scientific contribution, if applicable as different conventions apply per discipline, and that only authors have been added who made a meaningful contribution to the work.

Please note, we cannot investigate or mediate any authorship disputes. If you are unable to obtained agreement from all authors (including those who you wish to be removed) you must refer the matter to your institution(s) for investigation. Please inform us if you need to do this.

If you are not able to return a fully completed form within **30 days** of the date that it was sent to the author requesting the change, we may have to withdraw your manuscript. We cannot publish manuscripts where authorship has not been agreed by all authors (including those who have been removed).

Incomplete forms will be rejected.
